# Rayyan—a web and mobile app for systematic reviews

**DOI:** 10.1186/s13643-016-0384-4

**Published:** 2016-12-05

**Authors:** Mourad Ouzzani, Hossam Hammady, Zbys Fedorowicz, Ahmed Elmagarmid

**Affiliations:** 1 Qatar Computing Research Institute, HBKU, Doha, Qatar; 2 Cochrane Bahrain, Awali, Bahrain

**Keywords:** Systematic reviews, Evidence-based medicine, Automation

## Abstract

**Background:**

Synthesis of multiple randomized controlled trials (RCTs) in a systematic review can summarize the effects of individual outcomes and provide numerical answers about the effectiveness of interventions. Filtering of searches is time consuming, and no single method fulfills the principal requirements of speed with accuracy. Automation of systematic reviews is driven by a necessity to expedite the availability of current best evidence for policy and clinical decision-making.

We developed Rayyan (http://rayyan.qcri.org), a free web and mobile app, that helps expedite the initial screening of abstracts and titles using a process of semi-automation while incorporating a high level of usability. For the beta testing phase, we used two published Cochrane reviews in which included studies had been selected manually. Their searches, with 1030 records and 273 records, were uploaded to Rayyan. Different features of Rayyan were tested using these two reviews. We also conducted a survey of Rayyan’s users and collected feedback through a built-in feature.

**Results:**

Pilot testing of Rayyan focused on usability, accuracy against manual methods, and the added value of the prediction feature. The “taster” review (273 records) allowed a quick overview of Rayyan for early comments on usability. The second review (1030 records) required several iterations to identify the previously identified 11 trials. The “suggestions” and “hints,” based on the “prediction model,” appeared as testing progressed beyond five included studies. Post rollout user experiences and a reflexive response by the developers enabled real-time modifications and improvements. The survey respondents reported 40% average time savings when using Rayyan compared to others tools, with 34% of the respondents reporting more than 50% time savings. In addition, around 75% of the respondents mentioned that screening and labeling studies as well as collaborating on reviews to be the two most important features of Rayyan.

As of November 2016, Rayyan users exceed 2000 from over 60 countries conducting hundreds of reviews totaling more than 1.6M citations. Feedback from users, obtained mostly through the app web site and a recent survey, has highlighted the ease in exploration of searches, the time saved, and simplicity in sharing and comparing include-exclude decisions. The strongest features of the app, identified and reported in user feedback, were its ability to help in screening and collaboration as well as the time savings it affords to users.

**Conclusions:**

Rayyan is responsive and intuitive in use with significant potential to lighten the load of reviewers.

## Background

Randomized controlled trials (RCTs) play a pivotal role in medical research and are widely considered to be the best way of achieving results that can genuinely increase our knowledge about treatment effectiveness [1]. Although there is an increasing requirement for randomized controlled trials to guide healthcare decision-making, the synthesis of the results of more than one RCT in a systematic review can summarize the effects of their individual outcomes and provide numerical answers about the effectiveness of a particular intervention.

A systematic review is a summary of the medical literature that uses explicit methods to systematically search, critically appraise, and synthesize the data on a specific topic. The need for rigor in the production of systematic reviews has led to the development of a formal process for their conduct. This process has clearly designated steps to identify primary studies and the methods which will be employed to assess their methodological quality, the way in which data will be extracted, and the statistical techniques that will be used in the synthesis and reporting of that data [2]. Transparency and reproducibility are assured through the documenting of all of the decisions taken to include or exclude studies throughout the review process.

Identification of studies: The overarching aim is to ensure that an exhaustive scrutiny of the literature creates as comprehensive a list as possible of published and unpublished primary studies which are deemed relevant to answering the research question.

The number of citations generated by this search for eligible studies will depend on a variety of factors not least of all those involving some of the inherent aspects of the clinical topic. Thus, a clinical intervention which has been used extensively over a long period of time may be underpinned by a large body of research which in many instances may contain a substantial number of studies some of which may date back in excess of 20 years. Other possible contributory factors will include the comparative “interest” in the topic by clinicians, healthcare policy makers, and the media and may even include the potentially “vested” interest of the pharmaceutical industry.

Although the initial searches for trials for a systematic review may in some cases identify up to, and possibly extend beyond, 1000 citations, this will depend in part on the level of sensitivity and specificity built into the search strategy used to search the individual databases. While it is difficult to generalize what number of references to studies might be expected in an average yield, a minimum of 100 would not be an unreasonable number for many clinical topics.

Identification of potentially eligible studies: One of the most time consuming aspects of conducting a systematic review is the preliminary filtering or sifting through the citations from the searches, particularly if these number in the several hundreds and possibly in the thousands. Systematic review authors use a variety of electronic or manual methods to complete this task, which in any event must be double-checked by a co-author to ensure that all potentially eligible studies and those that require further full-text assessment have been identified. In addition, the tracking of decisions to include or exclude studies and the reporting of these judgments in a PRISMA flow diagram is mandatory for all Cochrane reviews and is now being done increasingly in other systematic reviews as this becomes a more widely accepted prerequisite for manuscript publication [3]. Moreover, the comprehensive documentation of these decisions by the review authors ensures the transparency, clarity, and traceability of the selection process and ultimately reinforces the robustness of the completed systematic review.

Identification and selection of studies can be challenging and very tedious, and a number of methods are used by review authors to facilitate the process. This can be performed either manually, i.e., by simply “highlighting” them in the printed copy of the search document by the use of different colors of a text marker, or electronically using the text highlighting function in the electronic copy of the search document. Alternative methods include the use of software such as EndNote or Reference Manager, if they are available to the review author. No single method can satisfactorily fulfill all the principal requirements of speed, accuracy, and simplicity in use, and each has its advantages, disadvantages, and adherents.

Interest in the automation of systematic reviews has been driven by a necessity to expedite the availability of current best evidence for policy and clinical decision-making as much as engaging with technology to allow review authors to redirect their focus on aspects where they are best at [4]. An increasing number of projects are underway which focus on the automation of segments of the systematic review process, and although several tools and software have been developed, so far, none of them span the entire process of review production [5].

Although the challenges faced by developers to automate and integrate the multiple steps in the workflow may seem insurmountable, recent advances in technology have helped overcome some of these hurdles [6]. However, accuracy and efficiency should not be sacrificed at the expense of speed, but flexibility, aligned with the potential for individual user customizability, should be built into the tool to allow for a range of users to create and use different personal preference-based interfaces. Automation should also target several key areas such as exploring ways of enhancing the user interface and user experience, developing systems which will ensure adequate workflow support, and the fostering of further developments in machine learning and data/text mining.

The process of automation of systematic reviews continues to present a number of additional challenges in that many of the tools have been developed independently as stand-alone software and are often not compatible with other tools [5]. In some instances, appropriate reliability and functionality testing has not been undertaken, and some tools are no longer being maintained by the developers or are prohibitively expensive to the average user. Moreover, some of the tools currently available require a level of technical skills beyond that of many review authors and also involve a steep learning curve and level of complexity which may necessitate a repetitive learn/re-learn phase if they are not used regularly. All of these challenges show how unsatisfying the existing landscape for systematic review automation is. The developers of Rayyan aim to address these challenges for providing an integrated solution, by working directly with systematic reviewers whilst continuously taking into account users’ feedback.

### Objectives

Rayyan (http://rayyan.qcri.org) was developed specifically to expedite the initial screening of abstracts and titles using a process of semi-automation but with a clear objective of incorporating a level of usability which would be compatible with the skillset of a broad cross-section of potential users. The ab initio objectives of the developers of the Rayyan app were to try and circumvent some of the complexities and challenges faced by reviewers with some of the existing tools. While our ultimate goal is to support the entire systematic review process, we initially focus on facilitating abstract/title screening and collaboration in addition to other supporting features around them. Thus, much of the focus of the development was on creating an inbuilt user-definable and partly self-customizable interface which would ensure Rayyan was largely intuitive in use as well as being user-friendly at all skill levels. We present here an exceptional case report of the development process of Rayyan, an app for the rapid exploring and filtering of searches for eligible studies for systematic reviews.

## Methods

There was a recognition by the developers of the need for a tool which would satisfy the requirements of a broad spectrum of review authors with a diverse range of competencies and skills and specifically one which would permit rapid and reliable exploration and sharing of search results but without being technologically burdensome. Therefore, engagement with an experienced Cochrane systematic review author (ZF) who had worked extensively with a large number of co-authors with mixed levels of experience proved to be pivotal to the development process.

The app underwent pilot testing prior to release and had extensive subsequent evaluation from a wide range of users, with a variety of skill levels and competencies, from across the globe. Sharing of user experiences and a reflexive response by the developers to an evolving “wish list” of requests by users enabled modifications and improvements to be made progressively and in real time, all of which proved to be a highly productive and effective collaboration in the development of Rayyan.

### Overview and architecture

**Fig. 1:**
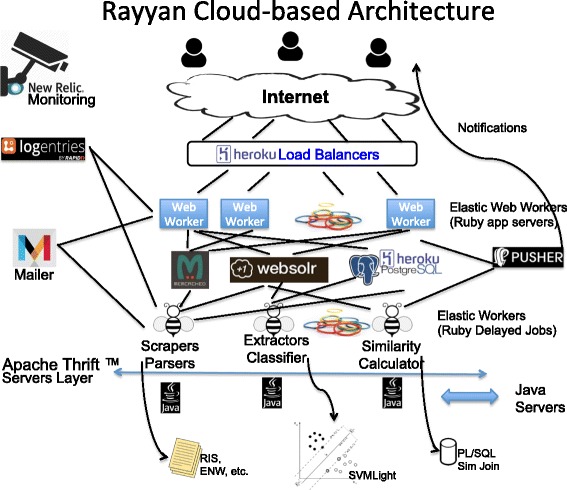
Rayyan architecture. Rayyan is a fully cloud-based architecture that uses a cloud platform as a service allowing elastic scaling of resources as we get more users and more requests. Rayyan’s workers are distributed using the load balancer to different app servers (Ruby web workers). These workers are elastic; they auto-scale based on traffic to guarantee minimal response time. For longer jobs or the elastic delayed jobs (the worker bees), such as upload parsing, similarity computation, and label predictions, they are handled through a queuing system. All workers have access to the storage layers: Postgres (for permanent storage), Solr (for indexing and searching), and Memcached (for caching results). Other parts of Rayyan, written in Java, are attachable to the jobs using an Apache Thrift service. Real-time notifications, on job completion or chat messages, for example, are delivered using Pusher, while other transactional information are delivered using the Mailchimp Mandrill service. All system activities are logged by Logentries and later backed up on AWS S3, while live instrumentation and monitoring is done by NewRelic

Rayyan is built on top of a cloud-based multi-tier service-oriented elastic architecture (Fig. 1). Scalability in Rayyan is underpinned by this cloud-based architecture which allows it to scale accordingly during peak times and as the number of users grows and they create more reviews and upload more citations. Moreover, at times, Rayyan may be actively processing data for tens of users or is just staying idle. The cloud-based architecture enables it to expand or shrink its hardware resources as needed. As a result, it is cost effective in idle times, with no costs incurred for resources not being used, and at the same time horizontally scales out in busy times easily. Part of the resources are only manually scalable, which means that Rayyan administrators will need to upgrade them as needed, for example, in increasing database storage needs, push notifications volume, and email messages volume. Other resources are automatically scalable to support the appropriate traffic in a cost-effective manner without sacrificing performance. This applies to web servers and background job workers.

Rayyan itself is written in the popular open-source framework Ruby on Rails [7], and runs on Heroku [8] which is a Platform as a Service based on the cloud-hosting Amazon Web Services. It integrates with other cloud services to fulfill the different tiers it requires. Examples of these services are Heroku Postgres [9] for SQL database management; Logentries [10] for central logging, tagging, and alerting; NewRelic [11] for app analytics, health monitoring, and alerting; Pusher [12] for real-time push notifications; and HireFire [13] for auto-scaling the app according to load.

### Workflow and user experience

**Fig. 2:**
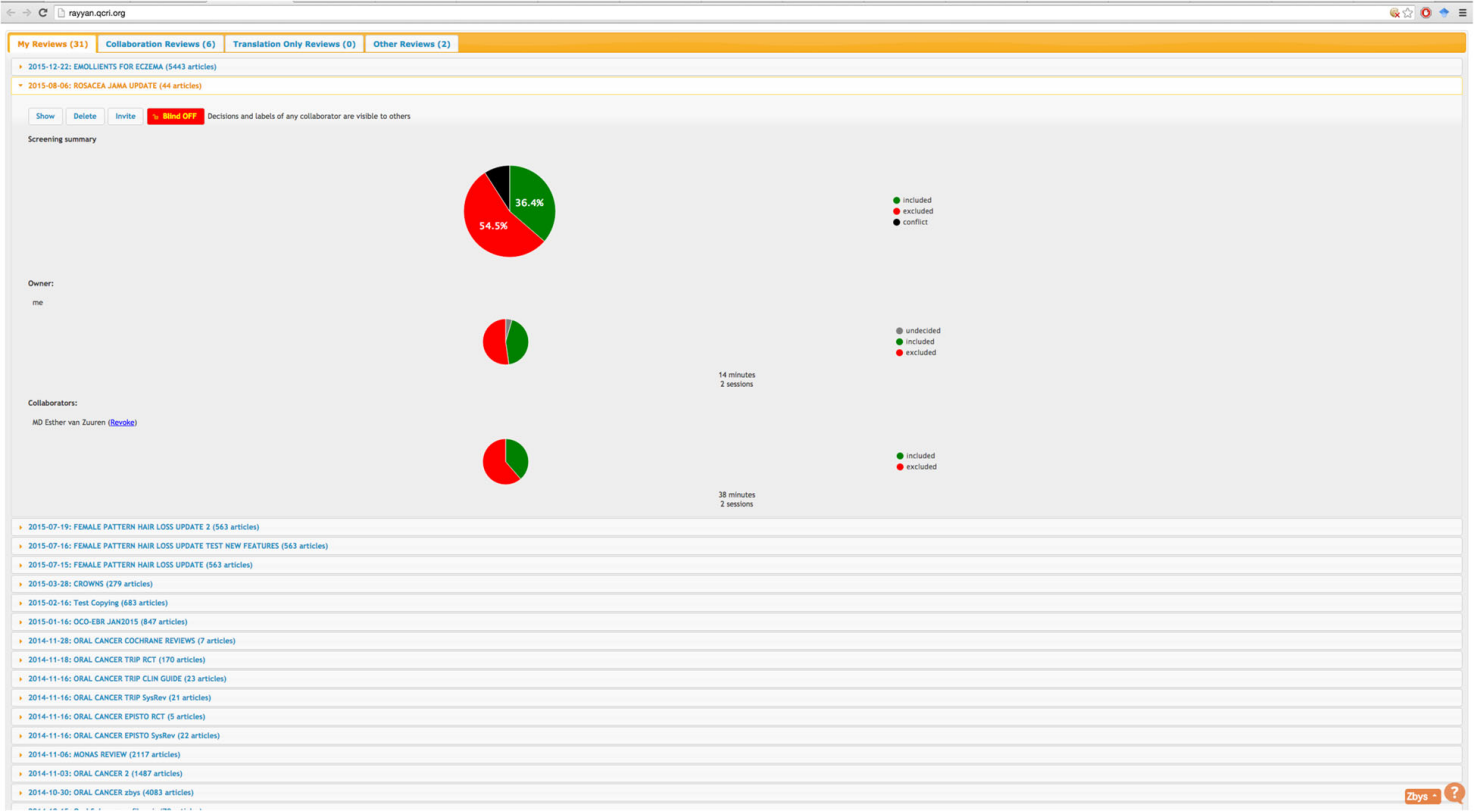
Rayyan dashboard. The dashboard lists all reviews for this user as well as for each review the progress in terms of decisions made and estimated time spent working on the review for all collaborators

**Fig. 3:**
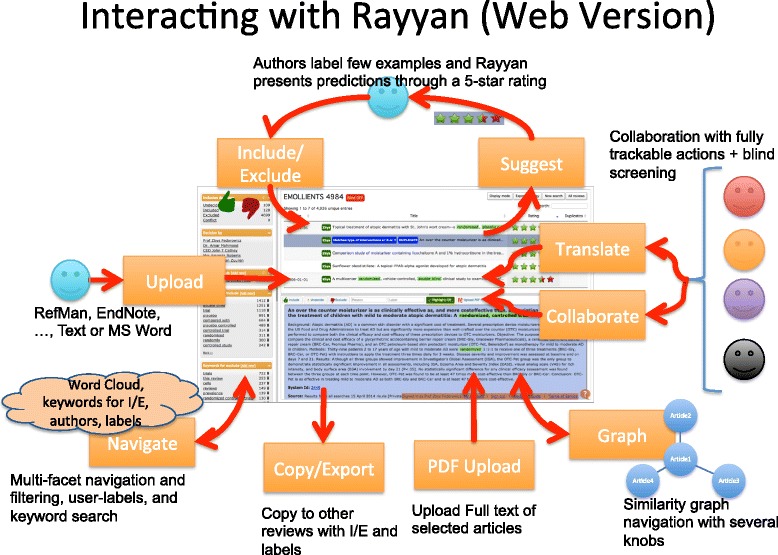
Rayyan workbench. The workbench shows the different ways users interact with the app

After logging into Rayyan, users are presented with a dashboard of all their current reviews (Fig. 2). They can either create a new review or work on an existing one. For each review, they upload one or more citation file obtained from searching different databases. Rayyan supports several standard formats, e.g., RefMan RIS and EndNote. At the outset, Rayyan processes the citation file by extracting different metadata, e.g., title, authors, and computing others, e.g., MeSH terms and language of the article, for each article or study in the citation file. These will then populate the facets in the review workbench (Fig. 3) to help explore and filter the studies. MeSH terms are presented as a word cloud allowing users to quickly grasp the main topics presented in the studies. In addition, users can filter studies based on two predefined lists of keywords that will most likely hint to either include or exclude a study. The user can also modify these two lists by removing and adding keywords, thus giving more flexibility in the labeling and selection of studies. Rayyan was seeded with two lists obtained from the EMBASE project to filter RCTs [14].

**Fig. 4:**
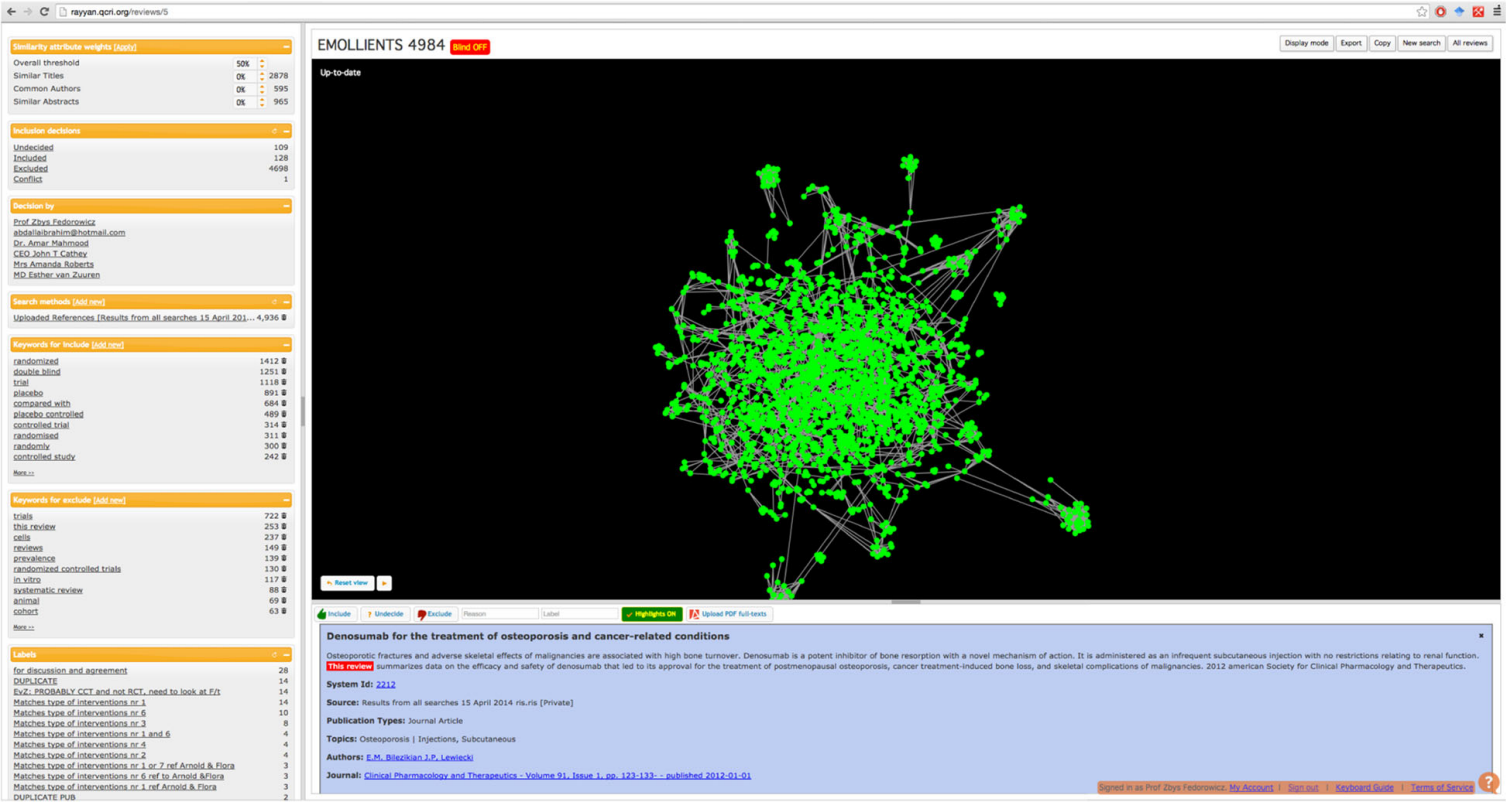
Similarity graph. Interacting with citations through the similarity graph

Users can also label their citations and define their individual reasons for exclusion which facilitates the sharing and tracking of these decisions. Citations can be explored through a similarity graph (Fig. 4) in which the citations are represented as nodes in a graph and clustered based on how similar they are (using an edit distance) in terms of title and abstract content as well as common authors. The similarity thresholds can be tuned independently for each attribute, i.e., title, abstract, and authors, as well an overall threshold.

### Rayyan mobile app

With the mobile app, users can screen reviews they have already uploaded from the web app. The most notable feature is the ability to use the app while offline. Users first download the entire review while online then work on it even in the absence of a network connection, and then, once connected, the app will automatically sync back to the Rayyan servers.

### Predicting included and excluded studies

An important feature of the Rayyan app is its ability to learn from users’ decisions to include or exclude studies which can then be used to build a model that would allow suggestions to be offered on studies that are awaiting screening. More specifically, after removing stop words and stemming the remaining words from the title and abstract, Rayyan extracts all the words (unigrams) and pairs of words (bigrams) and previously computed MeSH terms. These are then used as features by a support vector machine (SVM) classifier [15]. As users label citations to studies as excluded or included, Rayyan calls the SVM classifier which learns the features of these excluded and included citations and builds a model, or classifier, accordingly. The classifier then runs on the citations that await labeling and outputs a score of how close each study matches the include and exclude classes. That score is then turned into a five-star rating that is presented to the user. As the user continues to label more citations, if Rayyan believes it can improve its prediction quality, then it will use these new labeled examples to produce a new model and then run it on the remaining non-labeled citations. This process is repeated until there are no more citations to label or the model cannot be improved any further.

## Results and Discussions

### Evaluating the prediction algorithm

To test the quality of Rayyan’s SVM classifier, we used the above features on a collection of systematic reviews from a study published in [16]. In this study, test collections were built for each of 15 review topics (Table 1) which had been conducted by the Oregon EPC, Southern California EPC, and Research Triangle Institute/University of North Carolina (RTI/UNC) EPC. For each review, we know all the articles and what was included/excluded. The ratio of included articles ranged from 0.5 to 21.7%, with the largest review containing 3465 studies and the smallest 310.

**Table 1:** Statistics about inclusion and exclusion decisions for 15 systematic reviews from [16]

ID	Systematic review	Total	Abs	Full	%
1	ACEInhibitors	2544	183	41	1.61
2	ADHD	851	84	20	2.35
3	Antihistamines	310	92	16	5.16
4	AtypicalAntipsychotics	1120	363	146	13
5	BetaBlockers	2072	302	42	2.02
6	CalciumChannelBlockers	1218	279	100	8.21
7	Estrogens	368	80	80	21.7
8	NSAIDs	393	88	41	10.4
9	Opioids	1915	48	15	0.7
10	OralHypoglycemics	503	139	136	27
11	ProtonPumpInhibitors	1333	238	51	3.82
12	SkeletalMuscleRelaxants	1643	34	9	0.5
13	Statins	3465	173	85	2.4
14	Triptans	671	218	24	3.5
15	UrinaryIncontinence	327	78	40	12.2

A twofold cross-validation was used with 50% of the data going to training and 50% to testing. This process was repeated ten times, and the results were averaged. Two metrics were used for the evaluation of the quality of the classifier, AUC and WSS@95. The ROC (receiver operating characteristic) curve is obtained by graphing the true positive rate against the false positive rate as we vary the threshold used by the classifier. AUC refers simply to the area under this curve; 1.0 is a perfect score and 0.5 is equivalent to a random ordering. The work saved over random sampling measured at 0.95 recall (WSS@95), introduced in [16], refers to the percentage of studies that the reviewers do not have to go through because they have been screened out by the classifier at a recall of 0.95, compared to random sampling. ${\text {WSS}} = \frac {{\text {TN}} + {\text {FN}}}{N} - (1-{\text {Recall}})$ where TN is the number of true negatives, FN is the number of false negatives, and *N* is the total number of instances in the dataset. Recall refers to the recall of the positive class (included studies). The results we obtained are AUC=0.87±0.09 and WSS@95=0.49±0.18. The 49% result is important since it shows that Rayyan can help save time using the automatic prediction. While these results illustrate appreciable time savings for the prediction feature, it is important to keep in mind that Rayyan offers much more time savings because of all the facets, filtering features, and visual cues which help expediting the screening process.

### Pilot testing Rayyan

Pilot testing entailed the early evaluation of two specific aspects of functionality which had been built into the app. Critically important at the outset and before any further development could be considered was an assessment of how accurately Rayyan performed in a direct comparison with the manual methods which had been used on several Cochrane reviews. Equally significant, at this stage of the development process, was the necessity to provide the developers with an early overview of the potential added benefit of the “prediction” feature.

In December 2013, two Cochrane reviews, which had been authored and published previously by ZF, were used for the initial testing of the app [17, 18]. The search results for these two reviews, which were available as MS Word documents, provided references to 273 and 1030 individual studies. As these systematic reviews had already been published, the final selection of studies for inclusion and exclusion had been undertaken previously using “manual” methods (electronic highlight marker in the MS Word doc), and the consolidated results of the selection process were reported in the published Cochrane review. Tracking of the decisions at every stage throughout the selection process, including reasons for exclusion and agreements and disagreements between authors, had been annotated in the MS Word doc, and key details were reported in the PRISMA flow diagram in the published Cochrane review.

The testing phase commenced with the developers (HH/MO) creating separate folders for each of the Cochrane reviews in Rayyan followed by uploading of the corresponding searches for each review. Access (username/password) to the web site as well as an introduction to the functionality of the app was given to the tester (ZF) by the developer (HH). Although the “results” of the selection process were already known to the tester, and thus the experiment was not technically “blinded,” familiarity with the searches and the results at this stage allowed a quick overview of the look and feel of the app and enabled early comments by the tester on the functionality of the app which could then be proactively addressed by the development team.

The first and smaller of the “test” Cochrane systematic reviews (273 records) had been updated more recently, and the new searches and identified studies were already included in the latest version of the published Cochrane review. These additional searches for the update were subsequently uploaded into Rayyan, and the combined searches were subjected to further evaluation with the app but only after pre-testing of the earlier batch of searches. This Cochrane systematic review was used principally as a “taster” to allow the tester to become familiar with the app and to permit exploration of the options available for identifying, selecting, and tagging of the individual references using the Include/Exclude/Undecide “buttons” and to further annotate the reasons for exclusion if appropriate. All ad hoc responses and comments made by the testers and users during the early development phase were transmitted in real time through the “send us a message” function in the app such that these requests could be acted on contemporaneously by the developers and then re-evaluated further by the testers as part of an iterative process.

Testing of the Rayyan app on the second Cochrane review (1030 records) required a few attempts to identify the 11 trials which had been previously selected by the Cochrane review authors using “manual” methods during the process of conducting the systematic review. This part of the testing phase proved to be more substantive in view of the larger number of citations and also because it sought to assess the added value of the prediction features, i.e., “suggestions” and “hints.” These citations were star-rated (1 to 5 stars) based on a near-match similarity in text and wording and were offered to the tester as being potentially eligible studies for further consideration with the expectation that this would help in expediting the selection process.

### Testers’ comments

The testers’ initial comments indicated that overall the app was comparatively easy to use, readily navigable, and intuitive with no perceived requirement for a “Help” function. However, this option was discussed as a possible additional feature but which would be subject to the further “independent” and more extensive testing of the Rayyan app by a larger group of users.

**Fig. 5:**
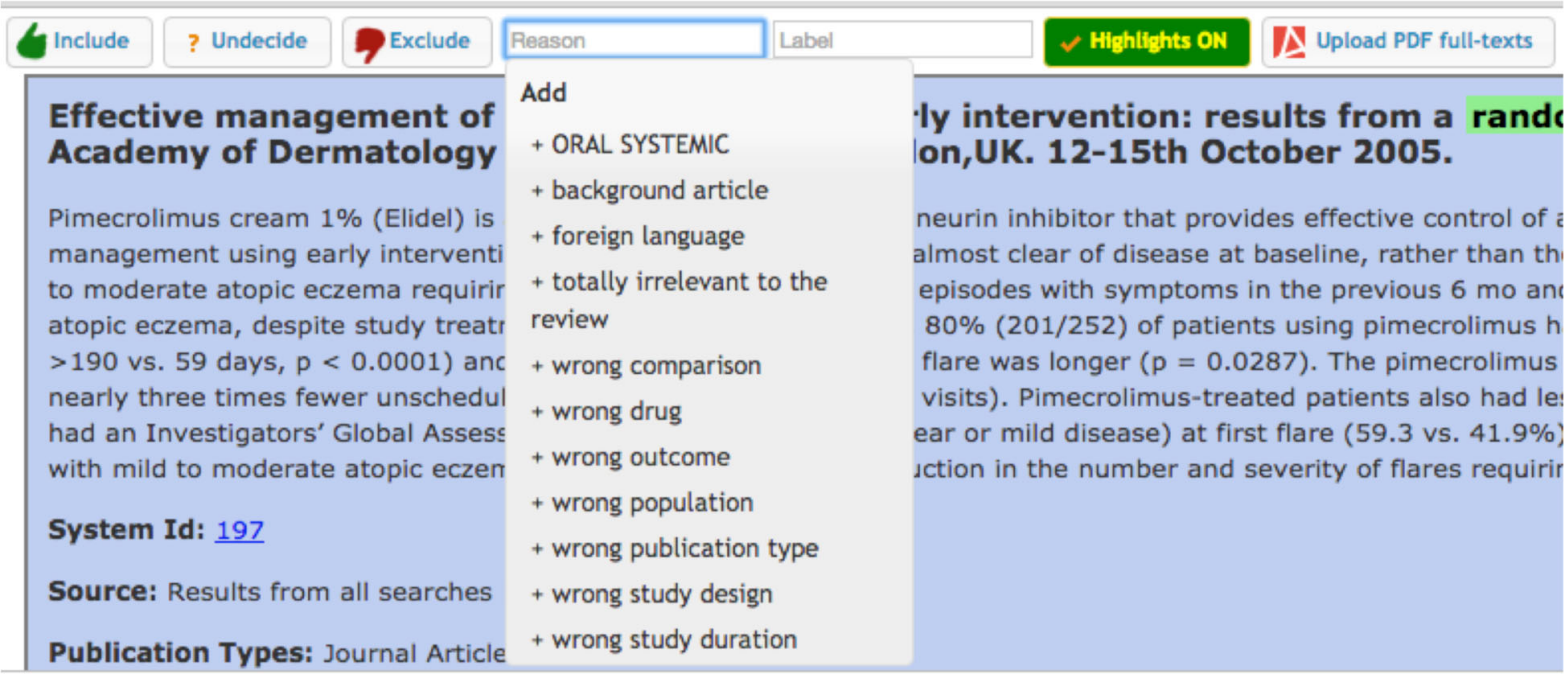
Reasons for exclusion. Users can select or add a reason for exclusion and exclude the study at the same time

**Fig. 6:**
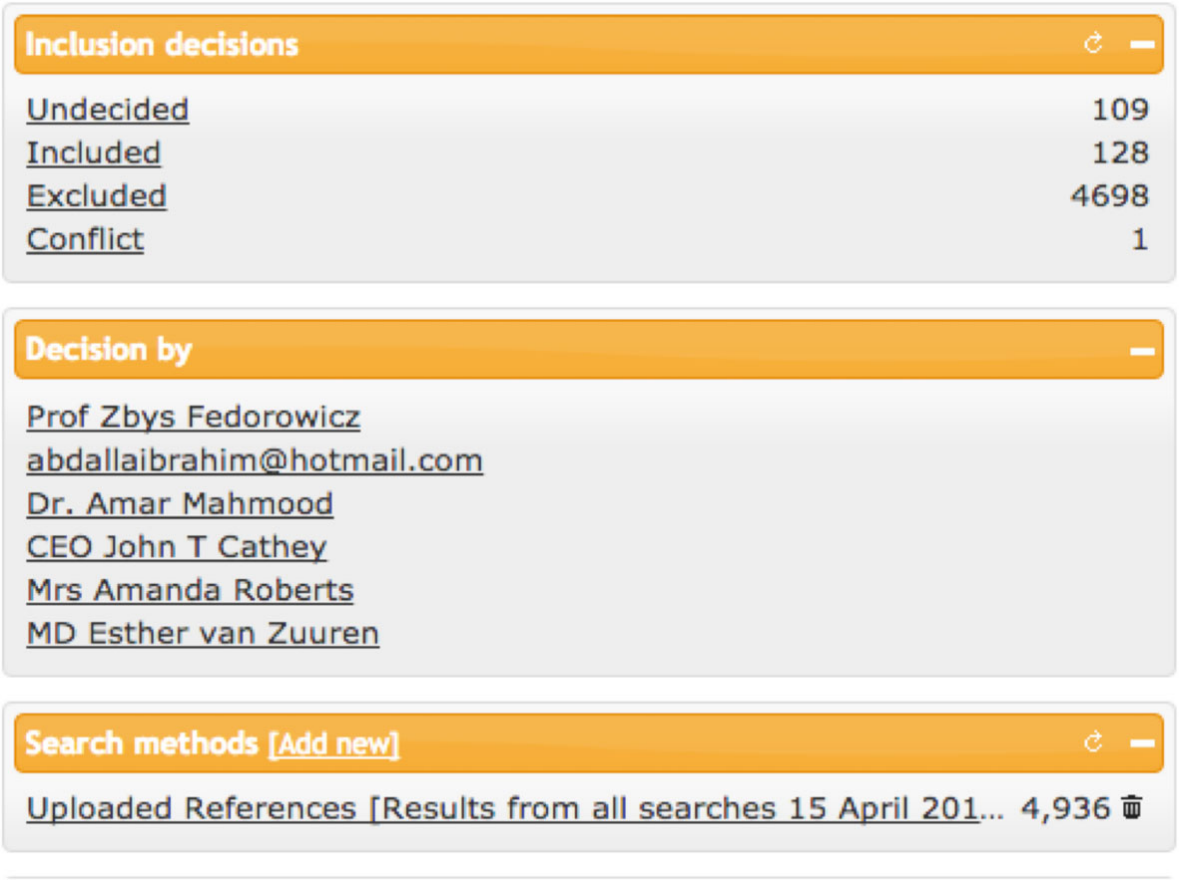
Filtering by exclusion/inclusion decisions by author

A number of key positive features were identified by the tester, as indeed were some areas that would require additional attention at this early stage of the development process. Particular reference was made to the immediate visibility of the selection options of “Undecided/Included/Excluded,” that they were one-click available which allowed for the quick tagging of studies and that these choices were clearly displayed, readily accessible, and immediately responsive on selection. Specific mention was made of the pulldown option under “Reasons” (see Fig. 5) which allowed the selection of either one or multiple generic and commonly used reasons why a study was to be excluded, i.e., “wrong population/ wrong publication type/wrong study design” but with the capability of adding other “self-generated” reasons to the existing predefined list. The capability of filtering the references by inclusion decision or by the collaborating author who made the decision provided an instantaneous overview of potential disagreements on study eligibility which could be discussed and resolved subsequently (see Fig. 6). The ability to quickly visualize the cumulative totals as studies were either excluded or included, and the word display of tagged studies which could be used as limiters were considered added-value functions. The topic summary word “cloud” was also noted in that it provided a very practical and graphical indication of the total number of studies identified by their keywords and with the number of studies correlating with the font size of the text in the word “cloud.”

Translation of study abstracts prior to assessment of a study for inclusion may be necessary if these have been published in other than the review author’s native language. A unique feature of Rayyan includes the option to be able to forward a link to the specific reference in the app directly to a selected translator who can then translate the portion of text or abstract and respond by pasting the translation directly below the study reference within the app. The ease and benefit of being able to do this directly within and from Rayyan were also highlighted in the initial testing phase. It was also noted in the early testing phase that some of the references to citations were incomplete and in some instances that the detail was substituted by a series of question marks replacing these details. This fault was reviewed by the developers and was considered to be due to errors incurred in formatting when the file was uploaded into Rayyan which could be readily identified and in general did not represent a significant number of references.

Testimonials from users highlighted the ease with which the exploration of searches could be completed, the large amount of time saved, the comparative simplicity and satisfaction in being able to readily share and compare individual authors’ decisions to include or exclude studies.

### Additional features incorporated after rollout

The highlighting of text, to enable rapid identification of important keywords, for example, trials and randomized placebo was considered by the developers and added as a “Highlights ON” button. Blinded and independent selection of studies is a critical aspect of the review process, and the option to be able to hide individual authors decisions about included studies was also added by request.

### User data after rollout

Rayyan has attracted significant interest from a large and well distributed number of users from around the globe. As of November 2016, there are more than 2000 users originating from more than 60 countries. These users are conducting hundreds of reviews on a total of more than 1.6M citations with the individual reviews ranging in size from tens to more than 38k citations.

### Workshops, presentations, and user feedback

Several opportunities arose in 2014/2015 to unveil Rayyan to the global research community, which included workshops at the Cochrane Colloquium in Hyderabad (2014), at Evidence-Live Oxford (2015), and the Cochrane Colloquium Vienna (2015). These expositions allowed for further development and the integration of several novel features based on feedback and suggestions received from attendees. We have also two other conduits through which users can give us feedback, a feature built into the web site and survey that our users can take any time (thus far, 66 respondents). From all of these feedback channels, the strongest feature of the app was its functionality, i.e., in the clear and unambiguous way in which studies could be viewed in context together with the completed selections, and how the “undecided” studies could be fed back into the system and that these were then highlighted as “hint.” From the survey, two important highlights relate to time savings and the most important features in Rayyan. Our users reported a 40% average time savings when using Rayyan compared to others tools, with 37% of the respondents reporting more than 50% time savings. For the second part, around 75% of the respondents mentioned that screening and labeling studies as well as collaborating on reviews as the two most important features of Rayyan.

### Future development

Based on the pilot study reported here and the different interactions with review authors, plans are underway to add several new features. The ultimate goal is to support most of the review process where machine learning, data/text mining, and information extraction techniques along with good software engineering best practices can provide clearly discernible quality coupled with speed, to facilitate reviewers efforts in the process of creating and updating systematic reviews. Key facets of the planned extensions include the following:

- Better detection of duplicates and a user-guided process for handling these duplicates.
- Assessment of risk of bias, with the initial focus on the domain-based criteria defined by Cochrane, to include identifying and extracting of supporting sentences from the full-text articles. Users will be able to validate these automatic judgments and annotate the full text with their own assessments.
- Automatic extraction of the values or the text related to PICO and other data elements. Again, users will be able to validate the extracted information and annotate the full text to extract more elements.
- Extending Rayyan API such that other software platforms can use Rayyan’s features by simple REST calls.

## Conclusion

Rayyan has been shown to be a very useful app with significant potential to lighten the load of systematic review authors by speeding up the tedious part of the process of selection of studies for inclusion within the review. Experiments on a set of 15 reviews showed that the prediction embedded in Rayyan can reduce the time for screening articles. In addition, our survey showed that our users reported time savings in the order of 40% on average compared to other tools they have been using in the past. Rayyan’s two most important features compared to other competitors are its ability to help in abstract and title screening and the ability to collaborate on the same review. A comprehensive comparison of Rayyan with other systems would require additional studies to be conducted, more especially those which build on several previous reports [7, 19]. These have been confirmed by our survey and the many testimonials from our users. Rayyan would benefit from several improvements including a better handling of duplicates, automatic data extraction from full text, automatic risk of bias analysis, and seamless integration with Review Manager (RevMan), the Cochrane software used for preparing and maintaining Cochrane reviews.

Rayyan is available for free at http://rayyan.qcri.org and is fully funded by Qatar Foundation, a non-profit organization in the State of Qatar.
